# 

**Published:** 1891-08

**Authors:** 


### LITERARY.

THE AMERICAN ELECTRO-THERAPEUTIC ASSOCIATION will hold its first annual meeting at the Hall of the College of Physicians, corner Locust and Thirteenth streets, Philadelphia, Pa., Thursday, Friday and Saturday, September 24th, 25th and 26th, 1891, under the Presidency of Dr. G. Betton Massey.

Physicians interested in the discussion of electricity in medicine, are invited to attend without further notice.

WM. H. WALLING, M. D., Secretary,
2005 Arch St., Philadelphia.

HORATIO R. BIGELOW, M. D.,
Chairman Executive Committee.

A COMPEND OF ANATOMY AND PHYSIOLOGY.—Edited and compiled by M. C. Tiers ; Fowler & Wells Co., Publishers, 775 Broadway.

This capital work has been compiled with special reference to the "New Model Anatomical Manikin," made and sold by the publishers, and will be found to be a great help to the student in his investigations of the elements and organs of physical life, without the inconvenience of more extended works. No possessor of the manikin should be without it—nor, indeed, any one who wants to get acquainted with himself at short hand.

IS MARRIAGE A LOTTERY ? By Evelyn Adams, 4to, 131 pp., price 25 cts.

We have read this book with a good deal of interest, but it does not solve the question. Indeed it would seem to picture marriage as little else than a lottery. A bull fight, with all the pomp and circumstance of those exciting diversions—a break down and headlong plunge into the mad arena of the idolized daughter of a Spanish Grandee—a bold dash and rescue by a young New Yorker—a subsequent accidental meeting—half hour of love-making and marriage engagement—an irate father ready to compromise at a $50,000 figure, at the end of a two years' probation—a cattle ranch venture and failure—a mining operation with no better luck, and finally a grand prize in a lottery and a dashing break for the aristocratic Don with the hard cash, just as he was about to retrieve his shattered fortunes by sacrificing his charming offspring, settled the business. Is marriage a lottery ? Of course it is !

VACATION TIME—with Hints on Summer Living. One of the series of "The Science of Health Library." Fowler and Wells Co., pub. Price 25 cts.

A timely volume filled with valuable information for travelers, tourists and campers.

CAUSE AND PREVENTION OF DIPHTHERIA.—An exhaustive report of a committee of five physicians, presented at the 18th Annual Meeting of the American Public Health Association. The next meeting of this association will be held at Kansas City, Mo., commencing October 10.

THE CENTURY MAGAZINE for July, in addition to its usual budget of good things, has a most instructive essay upon Abraham Lincoln, covering the period of the Civil War.

URICACIDŒMIA OXALURIA AND CYSTINURIA IN CERTAIN AFFECTIONS OF THE EYE.—By W. Cheatham, M. D., Louisville, Ky.

THE SOCIAL AND MEDICAL ASPECTS OF INSANITY.—By John Punton, M. D., Kansas City, Mo.

ANGELS' FOOD. A plea for Vegetarianism. By Basil Futcher, with many valuable suggestions quite in advance of the times.

